# Sustained HIV Viral Suppression 18 Months After the Last Dose of Long-acting Cabotegravir/Rilpivirine: A Case Report

**DOI:** 10.1093/ofid/ofaf399

**Published:** 2025-07-03

**Authors:** Shawnalyn W Sunagawa, Sara H Bares, Kimberly K Scarsi, Anthony Podany, Timothy Mykris, Jonathan Weinhold, Jennifer O’Neill, Maureen Kubat, Daniel Cramer, Jennifer M Davis, Joshua P Havens

**Affiliations:** Department of Pharmacy Practice and Science, College of Pharmacy, University of Nebraska Medical Center, Omaha, Nebraska, USA; Antiviral Pharmacology Laboratory, University of Nebraska Medical Center, Omaha, Nebraska, USA; Division of Infectious Diseases, Department of Internal Medicine, College of Medicine, University of Nebraska Medical Center, Omaha, Nebraska, USA; Department of Pharmacy Practice and Science, College of Pharmacy, University of Nebraska Medical Center, Omaha, Nebraska, USA; Antiviral Pharmacology Laboratory, University of Nebraska Medical Center, Omaha, Nebraska, USA; Division of Infectious Diseases, Department of Internal Medicine, College of Medicine, University of Nebraska Medical Center, Omaha, Nebraska, USA; Department of Pharmacy Practice and Science, College of Pharmacy, University of Nebraska Medical Center, Omaha, Nebraska, USA; Antiviral Pharmacology Laboratory, University of Nebraska Medical Center, Omaha, Nebraska, USA; Antiviral Pharmacology Laboratory, University of Nebraska Medical Center, Omaha, Nebraska, USA; Antiviral Pharmacology Laboratory, University of Nebraska Medical Center, Omaha, Nebraska, USA; Division of Infectious Diseases, Department of Internal Medicine, College of Medicine, University of Nebraska Medical Center, Omaha, Nebraska, USA; Division of Infectious Diseases, Department of Internal Medicine, College of Medicine, University of Nebraska Medical Center, Omaha, Nebraska, USA; Division of Infectious Diseases, Department of Internal Medicine, College of Medicine, University of Nebraska Medical Center, Omaha, Nebraska, USA; Division of Infectious Diseases, Department of Internal Medicine, College of Medicine, University of Nebraska Medical Center, Omaha, Nebraska, USA; Department of Pharmacy Practice and Science, College of Pharmacy, University of Nebraska Medical Center, Omaha, Nebraska, USA; Division of Infectious Diseases, Department of Internal Medicine, College of Medicine, University of Nebraska Medical Center, Omaha, Nebraska, USA

**Keywords:** antiretroviral therapy, HIV, long-acting cabotegravir/rilpivirine, pharmacokinetics, viral suppression

## Abstract

Long-acting cabotegravir/rilpivirine offers a low incidence of virologic failure. However, drug resistance can develop after discontinuation without an effective replacement regimen due to its long pharmacokinetic tail. We present a case of sustained virologic suppression 18 months after discontinuation of long-acting cabotegravir/rilpivirine despite inadequate adherence to oral antiretroviral therapy.

Long-acting cabotegravir/rilpivirine (LA CAB/RPV) is guideline recommended for the treatment of HIV; however, one key concern with its utilization is its long pharmacokinetic tail, particularly in the context of drug discontinuation [1–3]. This is a concern due to the potential for concentrations that are too low to prevent rebound viremia but high enough to exert drug pressure and select for HIV drug resistance in the absence of an alternative effective antiretroviral therapy (ART) regimen [1]. While LA CAB/RPV offers patients the option to no longer have to take daily oral ART to maintain virologic suppression, the pharmacokinetic tail adds additional complexity to patient care if patients are lost to follow-up or do not remain engaged in care. Here, we present a real-world case of LA CAB/RPV pharmacokinetics 18 months post discontinuation in a patient with chronic HIV who remained virologically suppressed despite significant gaps in oral ART adherence.

## CLINICAL CASE

A 33-year-old Hispanic male with chronic HIV infection presented to reestablish care with our clinic after relocating to another state 5 years prior. He had been diagnosed with HIV 7 years ago. HIV RNA at time of diagnosis was 44 230 copies/mL and peaked at 132 257 copies/mL immediately prior to starting ART. Baseline HIV-1 drug resistance RNA genotype (Genotype by Sequencing) was notable for wild type HIV without reverse transcriptase or protease inhibitor resistance. He enrolled in the FLAIR clinical trial [4], was randomized to oral dolutegravir/abacavir/lamivudine (DTG/ABC/3TC), and then transitioned to monthly LA CAB/RPV at week 100. He remained in the study but transferred to an out-of-state site and eventually transitioned to commercially available LA CAB/RPV (600/900 mg, intramuscular) dosed every 2 months. He continued taking LA CAB/RPV with his new HIV provider for approximately 10 months (ie, receipt of 5 doses) before discontinuing all ART, oral or injectable, on his own accord.

**Figure 1.**
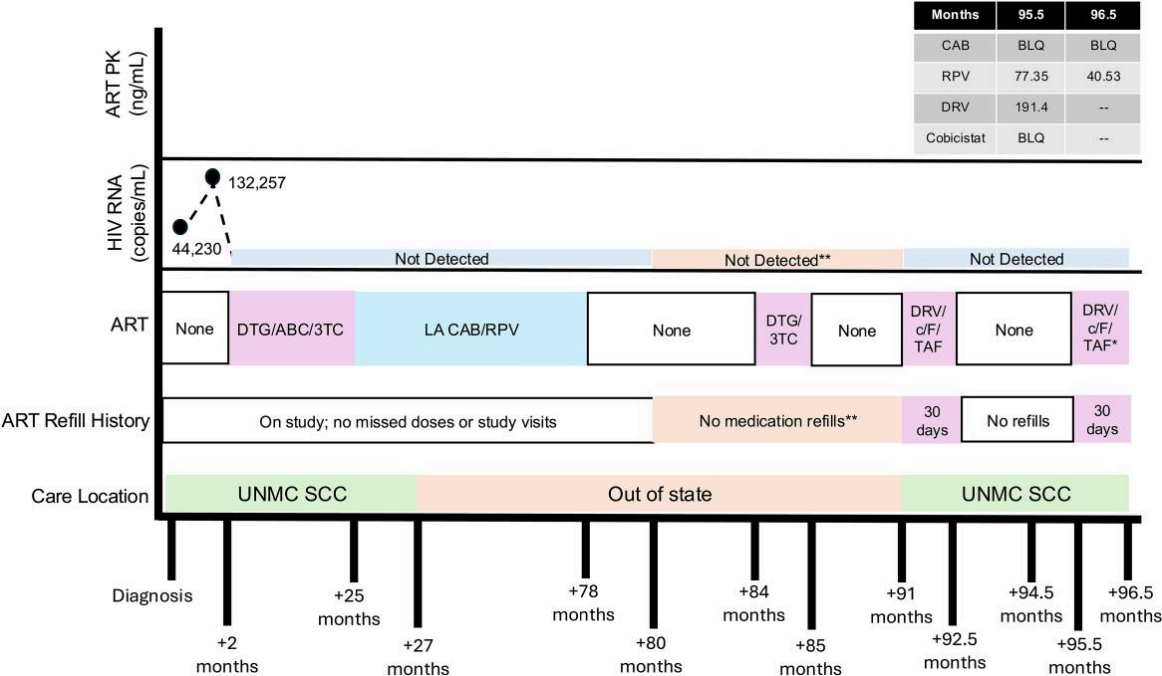
Antiretroviral, pharmacokinetic, and HIV RNA time course. *Patient reported intermittent adherence; last dose, 4 days prior to PK levels. **Out-of-state records confirmed with clinic providers. DTG/3TC prescribed, but the patient did not pick up the prescription. HIV-RNA remained undetectable. Abbreviations: 3TC, lamivudine; ABC, abacavir; ART, antiretroviral therapy; BLQ, below the limit of quantitation; c, cobicistat; CAB, cabotegravir; DRV, darunavir; DTG, dolutegravir; F, emtricitabine; LA, long acting; PK, plasma concentrations; RPV, rilpivirine; TAF, tenofovir alafenamide; UNMC SCC, University of Nebraska Medical Center Specialty Care Center.

He reestablished HIV care at our clinic 13 months later. He reported receiving a prescription for 1 month of DTG/3TC 6 months after his last LA CAB/RPV dose; however, he did not pick up the DTG/3TC prescription or other ART prescriptions after stopping CAB/RPV, and he reported not taking any other ART before reestablishing care. The prescription data were confirmed with his out-of-state providers and prescription refill registry (CyncHealth). He was initiated on darunavir/cobicistat/emtricitabine/tenofovir alafenamide (DRV/c/F/TAF) at his reestablishment visit due to presumed risk of HIV drug resistance. Figure 1 illustrates the ART timeline.

Laboratory tests at his reestablishment visit revealed unremarkable blood counts, chemistries, and lipids and a CD4 count of 980 cells/mm^3^ (41.9%). Additionally, his HIV RNA was undetectable, despite no oral ART for approximately 6 months prior to reestablishing care and 13 months from his last LA CAB/RPV dose. Given concern for possible resistance despite the currently undetectable HIV RNA, an HIV-1 drug resistance viral DNA genotype (GenoSure Archive) was obtained and was notable for a G190G/E mutation, indicating resistance to the nonnucleoside reverse transcriptase inhibitor (NNRTI) class of ART [5]. Selection of integrase strand transfer inhibitor drug resistance was not found. The patient was instructed to continue taking DRV/c/F/TAF with follow-up in 6 weeks to reassess HIV RNA.

At his 6-week follow-up visit, he reported perfect subjective and objective (Figure 1) adherence to DRV/c/F/TAF, although he voiced dissatisfaction with oral ART and a desire to switch back to LA CAB/RPV. Due to NNRTI resistance, he was instructed to continue taking DRV/c/F/TAF, and his HIV RNA remained undetectable. At his 3-month visit after reestablishing care, he reported not taking his DRV/c/F/TAF for approximately 1 month due to continued dissatisfaction with oral ART (Figure 1) and expressed curiosity about why he remained virologically suppressed while not taking any ART prior to reestablishing care. HIV RNA at this visit also remained undetectable despite no ART for the previous month. At this visit, a shared decision was made to restart his DRV/c/F/TAF with plans to return to clinic in 2 weeks to reassess adherence. At the 2-week follow-up (about 5 months after reestablishing care), we obtained plasma to measure concentrations of CAB/RPV and DRV/c (Figure 1).

Plasma concentrations of CAB/RPV were obtained approximately 17 and 18 months after the last dose of 600-mg intramuscular CAB and 900-mg intramuscular RPV. The concentrations 17 months after the last dose were below the limit of quantitation (BLQ) for CAB (lower limit of quantification, 20 ng/mL) and 77.35 ng/mL for RPV. The concentrations 18 months after the last dose were BLQ for CAB and 40.53 ng/mL for RPV. Plasma DRV and cobicistat concentrations were obtained approximately 4 days after the patient's last reported dose of DRV/c/F/TAF, following a 2-week period of inconsistent adherence prior to blood sampling. The concentrations were 191.4 ng/mL for DRV (median C_min_ at steady state, 1310 ng/mL [6]) and BLQ for cobicistat (lower limit of quantification, 20 ng/mL). After reeducation, medication counseling, and a discussion about potential oral and long-acting injectable ART options moving forward, the patient elected to remain off all ART due to sustained virologic suppression in the absence of ART with plans for quarterly HIV RNA monitoring.

## DISCUSSION

This case provides a unique real-world perspective on the long pharmacokinetic tail of LA CAB/RPV and the potential clinical challenges that may occur after abrupt discontinuation of LA ART. This case also highlights the growing need to better understand LA ART pharmacokinetic thresholds as they relate to virologic outcomes [7, 8].

Previous data support that to achieve HIV viral suppression, adherence to oral ART may need to be at least ≥90%, with newer data from integrase strand inhibitor–based regimens suggesting that adherence ≥75% may be sufficient to achieve virologic suppression [9, 10]. With LA CAB/RPV, daily adherence is replaced with the requirement of injection within the dosing window for each visit to ensure sufficient drug concentrations [11]. In our patient, oral ART adherence metrics were significantly below 75% to 90% when he was not actively receiving LA CAB/RPV, as confirmed subjectively by patient report and objectively with pharmacokinetic sampling (Figure 1). Although CAB concentrations were BLQ at 17 to 18 months since his last LA CAB/RPV dose, RPV concentrations were still 40.53 to 77.35 ng/mL, approximately 3.4 to 6.5 times the protein-adjusted IC_90_ (90% inhibition concentration; RPV, 12 ng/mL), indicating mostly therapeutic levels based on a proposed target of 4 times the protein-adjusted IC_90_ (48 ng/mL) [12, 13]. Despite these significant gaps in LA and oral ART adherence over 18 months, the plasma concentrations of DRV, CAB, and RPV support that the patient maintained virologic suppression with essentially RPV monotherapy despite evidence of RPV-specific drug resistance selection with archive genotype. Additionally, we acknowledge the potential boosting effect of concomitant cobicistat on the RPV concentrations; however, due to the timeline of ART administration (Figure 1), we do not believe that this significantly contributed to the sustained RPV concentrations observed 18 months after the last dose of LA CAB/RPV. Furthermore, the patient did not report any other potential concomitant medications that would have led to elevated CAB or RPV concentrations.

Previous data support a low incidence of virologic failure with LA CAB/RPV (approximately 1%–2%), although there is significant concern for potential rapid selection of resistance to integrase strand transfer inhibitor and/or NNRTI classes of ART following virologic failure [8, 14]. While our patient did not have virologic failure, archived proviral DNA genotyping revealed a new G190G/E mutation conferring resistance to the NNRTI class of ART, including RPV. We believe that this was an acquired new mutation with LA CAB/RPV, since his initial HIV-1 drug resistance RNA genotype (Genotype by Sequencing) at diagnosis revealed wild type HIV. Yet, we acknowledge that the usefulness of results from proviral DNA assays is still under investigation as it relates to utilization in a clinical setting [2]. This case emphasizes the important gaps in our understanding of the pharmacokinetic thresholds for LA ART required to prevent development of resistance, which can be associated with virologic failure.

This case demonstrates the complexity of sustained virologic suppression despite the patient's significant gaps in LA and oral ART. Potential contributors to the patient's sustained virologic suppression include residual LA ART concentrations, which may have been sufficient to suppress viral replication, and previous duration of sustained virologic suppression prior to discontinuing ART (Figure 1). Additionally, while the patient's initial HIV-RNA levels demonstrated that he was unlikely to be an elite controller, there is the possibility that he could be a posttreatment controller, which contributed to his ability to remain virologically suppressed without oral or LA ART [15]. Due to this sustained virologic suppression, the patient elected to remain off ART even though potential alternative options included restarting DRV/c/F/TAF, transitioning to bictegravir/emtricitabine/tenofovir alafenamide, or utilizing LA CAB/RPV in combination with subcutaneous lenacapavir. Given the patient's decision to remain off ART, we acknowledge the potential concern for virologic rebound with new ART resistance. Finally, we believe that this case emphasizes the importance of assessing the real-world pharmacokinetics of LA ART, especially its pharmacokinetic tail phase, to help better understand and define the pharmacokinetic thresholds as they relate to virologic outcomes.

## Notes

***Author contributions.*** All authors contributed to the reviewing and editing of the manuscript and approved the submitted version.

***Patient consent.*** The patient's written consent was obtained.

***Financial support*.** No financial support was provided relevant to this article.
